# KinCon: Cell‐based recording of full‐length kinase conformations

**DOI:** 10.1002/iub.2241

**Published:** 2020-02-06

**Authors:** Florian Enzler, Philipp Tschaikner, Rainer Schneider, Eduard Stefan

**Affiliations:** ^1^ Institute of Biochemistry and Center for Molecular Biosciences, University of Innsbruck Innsbruck Austria

**Keywords:** drug side effect, kinase conformations, kinase dimer, KSR, LKB1, MAPK, AMPK, MEK, molecular interaction, pseudoenzyme, pseudokinase, PKA, RAS, RAF, BRAF, CRAF, scaffolding function, STRADα, undruggable, biosensor, allosteric inhibitor, oncokinase, kinase drug efficacies

## Abstract

The spectrum of kinase alterations displays distinct functional characteristics and requires kinase mutation‐oriented strategies for therapeutic interference. Besides phosphotransferase activity, protein abundance, and intermolecular interactions, particular patient‐mutations promote pathological kinase conformations. Despite major advances in identifying lead molecules targeting clinically relevant oncokinase functions, still many kinases are neglected and not part of drug discovery efforts. One explanation is attributed to challenges in tracking kinase activities. Chemical probes are needed to functionally annotate kinase functions, whose activities may not always depend on catalyzing phospho‐transfer. Such non‐catalytic kinase functions are related to transitions of full‐length kinase conformations. Recent findings underline that cell‐based reporter systems can be adapted to record conformation changes of kinases. Here, we discuss the possible applications of an extendable kinase conformation (KinCon) reporter toolbox for live‐cell recording of kinase states. KinCon is a genetically encoded bioluminescence‐based biosensor platform, which can be subjected for measurements of conformation dynamics of mutated kinases upon small molecule inhibitor exposure. We hypothesize that such biosensors can be utilized to delineate the molecular *modus operandi* for kinase and pseudokinase regulation. This should pave the path for full‐length kinase‐targeted drug discovery efforts aiming to identify single and combinatory kinase inhibitor therapies with increased specificity and efficacy.

**Abbreviations**
AIM: auto‐inhibitory module
ALK: anaplastic lymphoma kinase
BCR: breakpoint cluster region protein
BTK: bruton's tyrosine kinase
CDKs: cyclin‐dependent kinases
c‐Met: c‐Met proto‐oncogene‐receptor tyrosine kinase
DFG: Asp‐Phe‐Gly motif
EGFR: epidermal growth factor receptor
F[1]: fragment 1
F[2]: fragment 2
FDA: food and drug administration
HER3: human epidermal growth factor receptor 3
JAKs: janus kinases
KinCon: kinase conformation
KSR1: kinase suppressor of Ras1
LKB1: liver kinase B1
MEK1: mitogen‐activated protein kinase
mTOR: mammalian or mechanistic target of rapamycin
PCA: protein‐fragment complementation assay
PDGFR: platelet‐derived growth factor receptors
PKAc: cAMP‐dependent protein kinase, catalytic subunit
PPI: protein–protein‐interaction
PTM: post‐translational modification
RAF: rapidly accelerated fibrosarcoma, kinase
RAS: rat sarcoma, GTPase
RET: proto‐oncogene tyrosine‐protein kinase receptor Ret
RTK: receptor tyrosine kinase
SRC: proto‐oncogene tyrosine‐protein kinase Src
STADα: STE20‐related kinase adaptor protein α
TRIB2: tribbles pseudokinase 2
VEGFR: vascular endothelial growth factor

## 1 TARGETING THE KINOME

**Figure 1:**
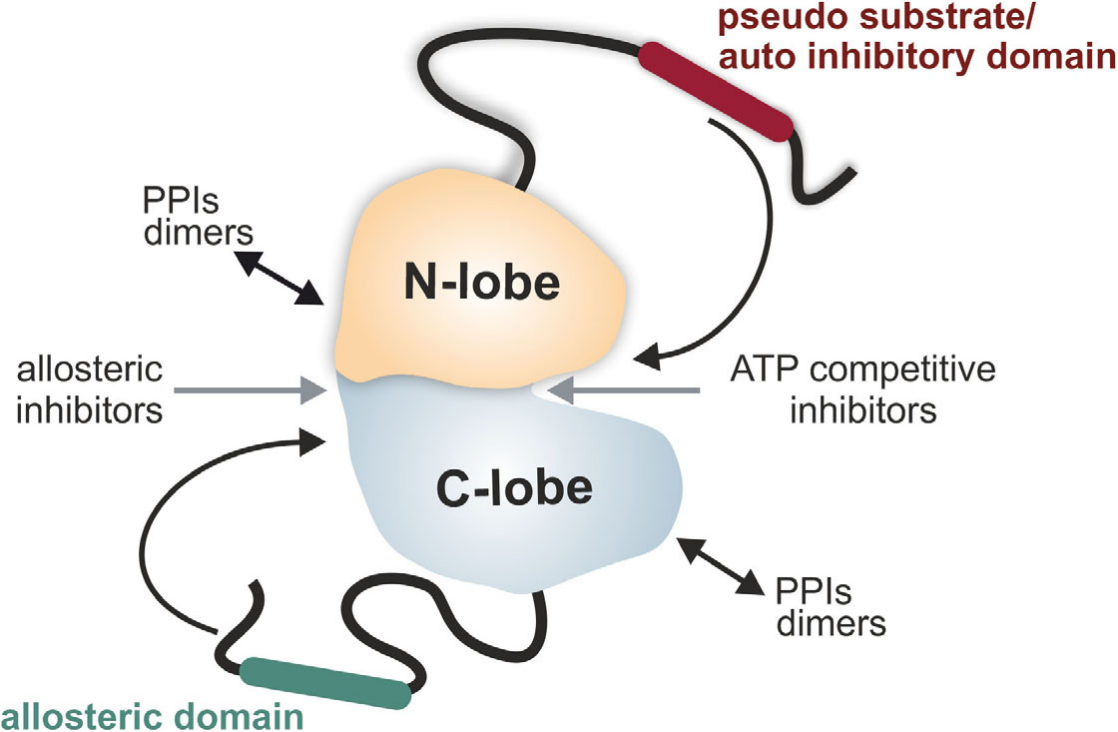
Kinase dynamics. Shown is a hypothetical full‐length kinase with cis‐regulatory motifs. The N and C lobes of the kinase core are centered. Different types of intra‐ and intermolecular interactions affect kinase activities and/or conformations. Allosteric kinase inhibitors/activators bind to the kinase domain. ATP‐competitive inhibitors bind into the catalytic cleft. Allosteric protein transformations occur through PPI, cis‐regulatory elements (e.g., auto‐inhibitory modules) or pseudosubstrate binding sequences which may promote activity‐related kinase conformation states

The human genome encodes more than 500 protein kinases that canonically catalyze the phosphorylation of compartmentalized protein substrates. Thereby, kinases act as molecular switches and central signaling hubs of the corresponding signaling cascade for propagating input signals. Kinases are at the heart of many signaling cascades, and therefore, it is no surprise that their activities are frequently deregulated in a collection of diseases due to mutations, overexpression, and/or transformations of their molecular interactions. Kinase dysregulation plays critical roles in the etiology and progression of many diseases including asthma, autoimmune, cardiovascular, inflammatory, nervous system diseases, and cancer.^1^, ^2^, ^3^, ^4^, ^5^ Approximately one quarter of all drug discovery efforts focus on the identification and refinement of lead molecules which target selected members of the kinase superfamily. So far, the US Food and Drug Administration (FDA) has approved more than 50 kinase inhibitors.^2^, ^6^, ^7^ Most of them target the ATP binding site of the kinase domain. The conserved protein kinase domain is the common functional core of all kinases. It consists of the N lobe which contains five strands of β‐sheets, and the C lobe which is mainly composed of α‐helices and loops. Recently, two N and C lobes connecting hydrophobic spines have been described. Upon structural alignment of key hydrophobic residues, the regulatory spine (R‐spine) represents the hallmark signature for an active kinase. The catalytic spine (C‐spine) is formed upon ATP binding.^8^, ^9^, ^10^ To fulfill phosphotransferase functions, these two lobes require conformational rearrangements for substrate binding, catalysis, and product release. Kinase inhibition targets the dynamic intrinsic properties of the kinase lobes and the R/C spines either through ATP‐competitive or allosteric small molecule inhibitors.^2^, ^11^ The current understanding of the dynamic nature of protein kinases in terms of structure and activation principles primarily involves this conserved kinase core.^12^ However, kinase functions further depend on the intra‐ and intermolecular communication through additional long‐distance allosteric effects (Figure 1). Due to missing full‐length kinase structures, the functional consequences of mutations or diverse types of molecular interactions remain poorly understood.

Conventionally, the quest for selective kinase inhibitors aims to identify chemical probes that are ATP‐competitive and lock the kinase domain in a specific inactive configuration. The active kinase core conformation is determined by the orientation of the so‐called DFG motif and the rotation of the αC helix. As a consequence a collection of inhibitors have been identified which target the distinct states of kinase‐domain conformations either in ATP‐competitive or allosteric fashion.^13^ FDA‐approved and ATP‐competitive kinase inhibitors are effective. Unfortunately, kinase inhibitor therapies include drawbacks which are related to cross‐reactivity and the occurrence of drug resistance mechanisms. Moreover, it needs to be noted that most clinical trials deal with the validation of inhibitors against kinase functions which are already targeted by existing inhibitors. Drug discovery efforts are still focusing on the well‐validated kinase target space despite of increasing evidence that also less frequently studied kinases are engaged in pathological cell functions.^3^, ^14^ There is a need to develop technologies to uncover and target unknown pathological kinase functions. In light of emerging concepts of precision medicine, drug discovery efforts for the screen of allosterically acting inhibitors should be promoted.^11^, ^15^ Such inhibitors possess the chance to be implemented into combination therapy approaches for increasing the specificity and efficacy of kinase inhibitor treatments.

Full‐length kinases are molecular switches that undergo conformational rearrangements that are tightly associated with their cellular functions.^16^, ^17^ Activity conformations of kinases oscillate and reflect alterations of the internal and external cell state or the respective signaling cascade.^18^, ^19^ The conformational plasticity of the kinase is central for the enzyme activation cycle. In the simplest way, kinase inhibition in *cis* is achieved by intramolecular binding of auto‐inhibitory module(s) or pseudosubstrate motifs to the kinase core.^20^, ^21^, ^22^, ^23^, ^24^, ^25^, ^26^, ^27^ Such conversions of protein conformations are triggered by diverse molecular interactions, competitive interactions, or post‐translational modifications. Moreover, kinase activity dynamics are influenced by the sensing of ligands, cofactors, and metabolites or by high‐affinity interactions with drugs. This involves diverse types of intermolecular protein–protein interactions (PPIs), kinase dimerization, and intramolecular protein dynamics (Figure 1).^1^, ^7^, ^18^, ^23^, ^28^, ^29^, ^30^, ^31^, ^32^, ^33^, ^34^, ^35^ Such facts are marginally considered in conventionally applied analyses of kinase functions or kinase inhibitor screens. Currently, a collection of cell‐free and cell‐based assays are available to identify the most promising kinase inhibitors.^36^ The underlying read‐outs primarily focus on the individual kinase domain or on the kinase‐mediated phospho‐transfer to the protein substrate.^37^, ^38^, ^39^, ^40^ Noninvasive cell‐based reporter assays for systematically studying the regulation, mode of action, and inhibition of full‐length kinases and their carcinogenic mutations are needed.^15^ We showed that in addition to the blocking of kinase activities, some ATP‐competitive kinase inhibitors change full‐length kinase conformations.^33^ We believe that some allosteric inhibitors transform conformation states as well. Therefore, we believe it is crucial to track the molecular motions of full‐length kinases directly in the proper cellular settings. Thus, easily adaptable and extendable biosensor technologies that allow the recording of intramolecular enzyme dynamics and drug‐driven transformations of pathological kinase conformations are needed.

## 2 KINASE CONFORMATION REPORTER

**Figure 2:**
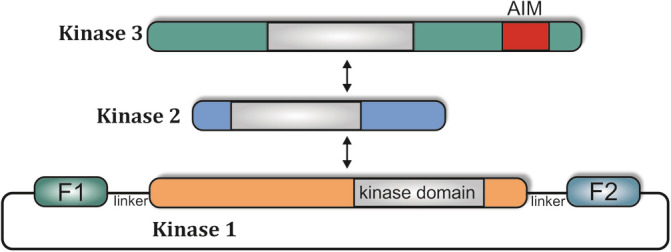
The adaptable and genetically encoded KinCon reporter. Modular structure of the KinCon reporter system. Mammalian expression vector encode for the full‐length kinase and flanked fragments of the luciferase PCA. A flexible linker separates F1 and F2 from the full‐length kinase which may contain additional cis‐regulatory elements. PCA, protein‐fragment complementation assay

We have recently generated a kinase conformation (KinCon) reporter platform for live‐cell measurements of full‐length kinase conformation dynamics.^33^ The adaptable KinCon biosensor system is genetically encoded and has a modular structure. The protein‐coding sequence of the respective kinase is embedded between two fragments (F [1] and F [2]) of a protein‐fragment complementation assay (PCA)‐based luciferase enzyme.^41^ Flexible linker sequences separate the PCA fragments from the kinase‐coding region. This allows the easy generation of new reporters for a wide variety of kinases by exchange of the respective kinase sequence (Figure 2).

**Figure 3:**
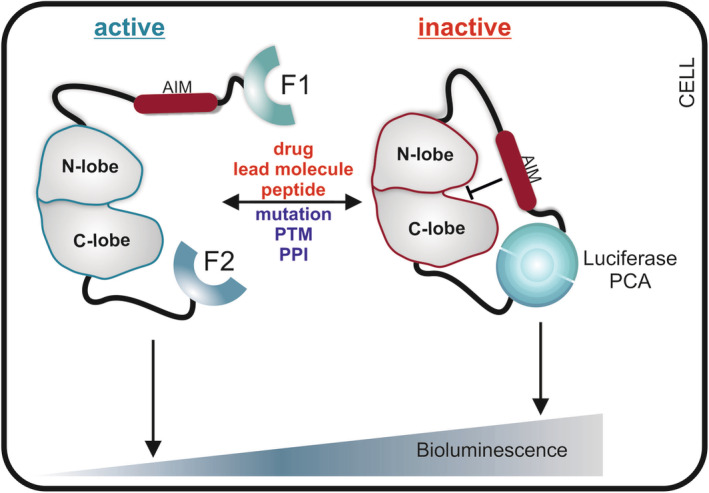
The KinCon biosensor system. Schematic depiction of the cell‐based KinCon reporter principle. A hypothetical kinase consisting of C lobe, N lobe, and AIM is indicated and fused to fragments 1 and 2 (F[1]/F[2]) of a luciferase PCA. We propose that the opened full‐length kinase conformation reflects the active one for many kinases (= ON state). The closed full‐length kinase conformation is the auto‐inhibited and inactive state (= OFF state) through the actions of an auto‐inhibitory module (AIM). Indicated means affect the opened, intermediate, or closed full‐length kinase conformations and may lead to an increase or decrease of luciferase‐PCA emitted cellular bioluminescence. PCA, protein‐fragment complementation assay

Different triggers activate or deactivate kinases which may lead to conformational rearrangements of the full‐length enzyme. Kinases are inactivated by kinase inhibitors, which mostly bind to the catalytic kinase cleft to inhibit the kinase in ATP‐competitive manner.^13^ Some kinases possess auto‐inhibitory regions to control their activity state.^31^ Other kinases contain amino acid stretches which act as pseudosubstrate motifs.^23^, ^42^ These modular domains interact in a context‐dependent way with the kinase domain and contribute to modulate kinase activities. Therefore, we assume that a collection of kinases engage an opened full‐length kinase configuration when the kinase is in the active ON state. Here, we present the KinCon reporter which is a bioluminescence‐based biosensor system for analyzing full‐length kinase conformation and their dynamics in the cell type of choice. Noninvasive KinCon measurements of kinase dynamics are performed in real time. It offers the possibility for customizing kinase reporter either by integration of patient‐mutations or by systematic modifications of PTM sites. Upon expression of the KinCon reporter in the appropriate cell line the biosensor engages an opened, intermediate or closed conformation. The opened and active full‐length kinase conformation (= kinase ON state) is adopted when the two fragments (F [1] and F [2]) of the PCA‐luciferase are spatially separated. In the presence of the luciferase substrate, less or no bioluminescence is emitted (Figure 3, left). In a more closed kinase conformation, the kinase is less active or inactive (= kinase OFF state). In this scenario, the two fragments are in close proximity to form a complemented and functional luciferase which catalyzes substrate conversion and consequently recordable light emissions^33^ (Figure 3, right). In the context of kinase catalysis, the open:closed KinCon reporter concept should not be mistaken with the kinase‐domain configuration. It describes the opened and closed orientation of N and C lobes in relation to each other thus reflecting the catalytic state of the kinase domain exclusively.^43^ A collection of different factors such as mutations, different kinds of molecular interactions, PTMs, lead molecules, approved kinase drugs, or substrate sequence delineated peptides may affect full‐length kinase activity conformations. In this context, time‐ and dose‐dependent lead molecule exposures bear the chance to record activity relevant full‐length kinase conformations and this in intact cells. Furthermore, these cell‐based assays have the potential to take cell‐type specific PPIs, PTMs, and the mutational kinase/PPI profile into account. Previously, we have shown that different cancer‐causing mutations affect conformational changes of one of the most frequently mutated kinases in melanoma, the oncokinase BRAF.^33^ In this proof of principle study we showed evidence that the effectiveness of FDA‐approved drugs targeting different BRAF kinase‐mutants can be anticipated. Furthermore, we have reassured the opened and closed RAF kinase conformation model^31^ using in vivo recordings of full‐length and mutated KinCon reporters. Thus, we unveiled unexpected allosteric effects of mutation‐specific anticancer drugs on the molecular interactions of the mutated BRAF oncoprotein which has implications for the architecture of a tetrameric RAS:RAF complex.^31^, ^33^

## 3 TRACKING KINCON ACTIVITY DYNAMICS

Despite the fact that only a small fraction of the kinome has been part of drug discovery efforts, so far more than 50 kinase inhibitors have been approved.^2^ Most of them target central oncokinases such as ALK, BCR‐Abl, BRAF, BTK, CDKs, c‐Met, EGFR family, JAKs, MEK, PDGFR, RET, Src family, and VEGFR.^11^ The search for non‐ATP‐competitive kinase inhibitors of these kinase families would open new possibilities for efficient combination therapy with the hope to overcome different types of frequently occurring kinase inhibitor‐drug resistance mechanism.^44^, ^45^ An advanced screening approach using KinCon reporters should allow identifying lead molecules which interfere with activity conformations in a cell‐type specific setup and at expression levels similar or below to the endogenous kinase. Given that with this method full‐length kinases can be tested, one would expect to catch also unique allosteric modulators of kinase functions using KinCon biosensors. The following matters need to be taken into account for generating a functional KinCon reporter: First, there is definitely a size restriction. Most kinases contain additional domains with diverse types of functions. The generation of KinCon reporters, which are based on a multi‐domain polypeptide chain might be challenging. Second, not for every kinase the simplified opened‐closed kinase concept will apply. Third, tagging might interfere with kinase function, molecular interactions, and/or the localization which should be determined. Fourth, membrane‐spanning domains as found in receptor tyrosine kinases (RTKs) might complicate the generation of a functional KinCon. Thus, one could envision to tag RTKs internally with specific luciferase‐PCA fragments. So far, we have published evidence that patient‐mutations and lead molecules interconvert full‐length BRAF kinase conformations.^33^ Recently, we demonstrated that KinCon dynamics can be tracked with KinCon reporter using MEK1 and the catalytic subunits of PKA (PKAc) as well (Mayrhofer et al., submitted). In addition, we show basal KinCon conformations with kinases from the mTOR pathway, some CDKs, and with kinases upstream of AMPK. It should be noted that the KinCon reporter principle may also provide functional insights into pseudokinase conformation dynamics.^34^, ^46^, ^47^, ^48^ Recently, it has been demonstrated that nucleotide binding, substrate interactions, or small molecule interactions alter non‐catalytic protein‐interaction modules of pseudokinase.^49^ These factors may lead to reorganization of the complete pseudokinase‐containing polypeptide, as it has been exemplarily described with the pseudokinase TRIB2.^34^, ^50^ Recent studies underline that there are remarkable parallels between pseudokinase modulation and their active counterparts.^46^ It is the intra‐ and intermolecular configurations of the kinase which contributes with and without phosphotransferase activities to the allosteric regulation of signal transmission. We propose that integrating KinCon into a medicinal chemistry screening approach could deliver chemical entities which lock both, kinase and pseudokinase in a less active state and conformation. Moreover, the accessibility of a putative nucleotide binding pocket in the different pseudokinases will foster the rational design of leads to interfere with kinase conformation plasticity. The specificity and efficacy of such lead molecules could be tested in intact cells and in a high throughput format using KinCon biosensors.

It is evident from the literature that pseudokinases and kinases functionally interact. In addition to phosphotransferase activities, the non‐catalytic kinase conformations/states participate in promoting signal propagation.^51^, ^52^ A collection of kinase:pseudokinase interactions have been unveiled which are crucial for signal transmission. Deregulation of such signaling pairs have been linked to the etiology of cancer. The pharmaceutical targeting of oncogenic kinase:pseudokinase units is challenging.^4^, ^34^, ^46^, ^47^, ^48^ One therapeutic target, which has been observed to be deregulated in a wide variety of human cancers, is the pseudokinase and human epidermal growth factor receptor 3 (HER3). HER3 upregulation is linked to several cancer types, in which it promotes tumor progression through its interaction with different catalytically active, membrane organized, and druggable RTKs. Two pseudokinases, which are suitable for KinCon and prone to become drug targets, are KSR1 and STRADα. KSR1 acts as molecular scaffold by interacting with kinases components of the RAS–RAF–ERK cascade. By orchestrating the interactions with RAF and MEK kinases, it is assumed that it enhances MAPK activation. It is controversial if a weak catalytic KSR1 activity is physiologically relevant. Given its broad implication of the RAS–RAF–ERK cascade in oncogenic signaling^53^ and the occurrence of drug resistance upon one‐way kinase inhibitor therapies,^44^ the combinatory targeting of scaffolding functions of KSR might improve therapy success. In this context, it is of interest that KSR binds cations and nucleotides which would allow to implement ATP‐competitive inhibitors.^46^

Another example which might be suitable for the KinCon reporter system is the interaction of a hetero‐trimeric complex consisting of the kinase LKB1, the interjacent scaffolding protein MO25a, and the pseudokinase STRADα. The concept of opened and closed full‐length pseudokinase conformations has been demonstrated for STRADα.^54^ LKB1 is a tumor suppressor and inactivating mutations cause the inherited Peutz–Jeghers cancer syndrome.^55^ LKB1 is the key upstream activator of multifaceted AMP‐activated protein kinase (AMPK) activities. Both kinases control cell growth in response to environmental nutrient changes. It has been shown that STRADα binds LKB1 as a pseudosubstrate.^54^ Formation of the trimeric complex results in LKB1 mediated phosphorylation and activation of AMPK. Thus, the scaffolding activity of STRADα is relevant for controlling LKB1 activity. The pseudokinase STRADα binds nucleotides in the absence of cations.^46^ The question arises how ATP‐competitive STRADα KinCon dynamics may affect wild type and mutated LKB1 functions. This would be a unique opportunity for combining pseudokinase and kinase KinCon reporter profiling experiments. Thus, we envision to use combinations of KinCon biosensors for systematic predictions of lead molecule efficacies targeting kinase or pseudokinase functions. This could unveil mechanistic details on interacting kinase protomer functions and open a drug discovery pipeline for (pseudo)kinase‐targeted combinatory therapy with hopefully increased specificity and efficacy.

## CONFLICT OF INTEREST

The KinCon reporter is subject of patent applications (E.S.; University of Innsbruck).
